# The Nutrient-Sensing Hexosamine Biosynthetic Pathway as the Hub of Cancer Metabolic Rewiring

**DOI:** 10.3390/cells7060053

**Published:** 2018-06-02

**Authors:** Ferdinando Chiaradonna, Francesca Ricciardiello, Roberta Palorini

**Affiliations:** Department of Biotechnology and Biosciences, University of Milano-Bicocca, Milan 20126, Italy; francesca.ricciardiello@unimib.it (F.R.); roberta.palorini@unimib.it (R.P.)

**Keywords:** hexosamine biosynthesis pathway, UDP-Glc*N*Ac, protein glycosylation, metabolism, bioenergetics, cancer

## Abstract

Alterations in glucose and glutamine utilizing pathways and in fatty acid metabolism are currently considered the most significant and prevalent metabolic changes observed in almost all types of tumors. Glucose, glutamine and fatty acids are the substrates for the hexosamine biosynthetic pathway (HBP). This metabolic pathway generates the “sensing molecule” UDP-*N*-Acetylglucosamine (UDP-Glc*N*Ac). UDP-Glc*N*Ac is the substrate for the enzymes involved in protein *N*- and *O*-glycosylation, two important post-translational modifications (PTMs) identified in several proteins localized in the extracellular space, on the cell membrane and in the cytoplasm, nucleus and mitochondria. Since protein glycosylation controls several key aspects of cell physiology, aberrant protein glycosylation has been associated with different human diseases, including cancer. Here we review recent evidence indicating the tight association between the HBP flux and cell metabolism, with particular emphasis on the post-transcriptional and transcriptional mechanisms regulated by the HBP that may cause the metabolic rewiring observed in cancer. We describe the implications of both protein *O*- and *N*-glycosylation in cancer cell metabolism and bioenergetics; focusing our attention on the effect of these PTMs on nutrient transport and on the transcriptional regulation and function of cancer-specific metabolic pathways.

## 1. Introduction

Cancer cell growth is tightly correlated to an altered cellular metabolism. In particular, as Warburg noticed almost one century ago, cancer cells, even in the presence of oxygen, produce Adenosine Triphosphate (ATP) through glycolysis, a catabolic pathway predominantly considered to be of great importance under anaerobic conditions and that produces much less ATP than oxidative phosphorylation [1]. Nevertheless this alteration, known as the “Warburg effect”, is nowadays considered a cancer hallmark, since it actively participates in the increasing requirements for macromolecular synthesis necessary to maintain rapid cancer proliferation. Indeed, the increased glucose consumption is used as carbon source for the de novo generation of nucleotides, lipids and proteins by its redirection into the multiple branching pathways that originate from glycolysis. It has been shown that increased glucose uptake allows a high rate of nucleotides and of reducing equivalents synthesis (NADPH) through the pentose phosphate pathway (PPP) [2,3], which are then used respectively to sustain DNA replication and mRNA transcription and most notably for lipid synthesis. In addition, glycolytic flux is also redirected into serine biosynthesis, since serine is a precursor of the nonessential amino acids glycine and cysteine. Glycine is in turn a precursor of purine nucleotide bases, and together with cysteine, of glutathione (GSH). Serine is also necessary for the production of sphingolipids and phospholipids and supplies carbon to the one-carbon pool, which is involved in folate metabolism, that participates in thymidine synthesis and the regeneration of methionine from homocysteine and thus facilitates the generation of *S*-adenosylmethionine (SAM), the methyl donor for both DNA and histone methylation. On the other hand, the pyruvate produced by glycolysis is converted to lactate, and this step is essential for the regeneration of NAD^+^ in the cytosol to sustain the high glycolytic rate observed in tumors [2,3]. Therefore, this metabolic switch from oxidative respiration to glycolysis is indispensable, providing cancer cells with ATP and most of the anabolic intermediates required to sustain high rates of cellular proliferation, in terms of nucleotide synthesis, reducing power (NADPH, NADH) and building blocks for lipid and amino acid biosynthesis.

**Figure 1:**
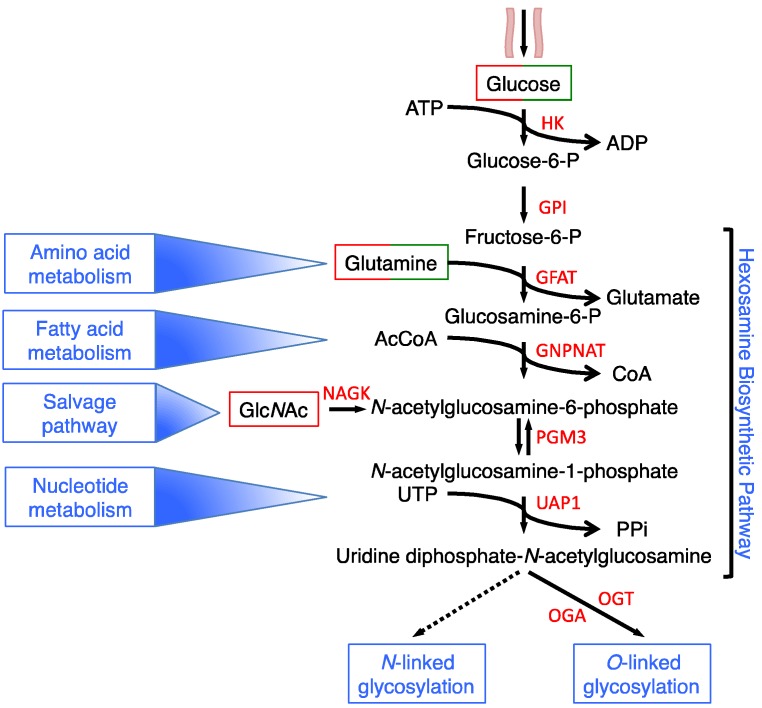
Schematic representation of the hexosamine biosynthesis pathway. HBP enzymes are depicted in red, metabolites in black, cellular processes in light blue. The green/red boxes indicate the metabolites whose change induces UDP-Glc*N*Ac decrease (green) or increase (red).

Another branch of glycolysis, in which the glucose is directly diverted, is the hexosamine biosynthesis pathway (HBP) [4,5]. The HBP is characterized by six enzymatic steps, of which two are shared with glycolysis (Figure 1).

In fact, hexokinase (HK) phosphorylates glucose to produce glucose-6-phosphate, which is then converted into fructose-6-phospate by phosphoglucose isomerase (GPI). Fructose-6-phospate and glutamine are converted into glucosamine-6-phosphate (GlcN-6P) and glutamate (Glu) by the first HBP rate-limiting enzyme glutamine: fructose-6-phosphate transaminase (GFAT). The next enzyme, glucosamine-phosphate *N*-acetyltransferase (GNPNAT) converts the GlcN-6P and the Acetyl-Coenzyme A (Ac-CoA) into *N*-acetylglucosamine-6-phosphate (Glc*N*Ac-6P) and CoA. Then, Glc*N*Ac-6P is isomerized in Glc*N*Ac-1P by the Glc*N*Ac phosphomutase (PGM3/AGM1) and finally, the UDP-*N*-acetylglucosamine pyrophosphorylase (UAP1) converts UTP and Glc*N*Ac-1P into UDP-*N*-acetylglucosamine (UDP-Glc*N*Ac) and pyrophosphate (PPi). UDP-Glc*N*Ac is the substrate for *O*- and *N*-glycosylations, being a component of simple and complex branched *O*- and *N*-glycans, whose attachment to the proteins leads to the formation of complex glycoproteins and proteoglycans named glycoconjugates. These are mainly expressed on the cell surface, where they form a dense array, the so-called glycocalyx, or are secreted into the extracellular space where they are incorporated into the extracellular matrix [6]. UDP-Glc*N*Ac is also the substrate for a less complex type of glycoconjugate, the protein *O*-Glc*N*Acylation [7], which has been found on many cytoplasmic, nuclear and mitochondrial proteins, and is considered a dynamic PTM, analogous to phosphorylation, regulating many cellular functions [8]. Glycan structures and types depend on the gene expression, on the activities of several enzymes, i.e., the enzymes present in the Golgi, and on the availability of the main donor substrate, UDP-Glc*N*Ac.

Although the glucose flux is possibly different in the various cell types, some reports have indicated that around 1–3% of total cellular glucose is channeled into the HBP [9]. Several data underline the role of the HBP as an integrator and sensor of the main cellular metabolic molecules, namely carbohydrates (glucose), amino acids (glutamine), lipids (Acetyl-CoA, Ac-CoA) and nucleotides (uridine), in order to synthesize the final product of the pathway UDP-Glc*N*Ac. This metabolite is not directly involved in any anabolic process, since it fundamentally serves, as previously described, as a building block for protein- and lipid-glycoconjugate biosynthesis [5,6,7,10]. In this context, the ability of HBP to integrate and sense the main intracellular metabolites strongly supports the important role of this pathway and of its final product UDP-Glc*N*Ac in the regulation of cell metabolism both in physiological conditions and in the metabolic changes observed in several human diseases, such as cancer. Therefore, in this review we explore the connection between the HBP and cell metabolism with a particular emphasis on the association between HBP and the metabolic rewiring observed in cancer cells.

## 2. HBP Flux Is Modulated in a Nutrient-Dependent Fashion

Several authors have shown that changes in intracellular nutrient concentration modulate the intracellular UDP-Glc*N*Ac amount and HBP flux (Figure 1). In this regard it has been shown that increased glucose availability in isolated adipocytes, pretreated with insulin to enhance their glucose transport, induces a rapid increase of UDP-Glc*N*Ac, since this increase can be seen as early as 5 min after the addition of glucose and remains almost stable until 4 h [11]. Similar results have been obtained in different cell models, such as mesangial cells [12], colon cancer cells [13] and human cervix cancer cells [14]. Conversely, 24 h of glucose depletion in different types of hematopoietic cells induces an early reduction of UDP-Glc*N*Ac [15]. Comparable results have been also obtained in K-Ras-transformed mouse fibroblasts [16], human hepatocellular carcinoma cells [17], breast cancer cells and pancreatic cancer cells [18,19]. Notably, it has also been shown that glucose reduction may also enhance the HBP flux, in particular protein *O*-Glc*N*Acylation, by increasing the mRNA and the protein expression of some HBP enzymes. In fact, some authors have reported the capacity of different types of cells to activate compensative mechanisms to sustain HBP flux and survival under moderate glucose depletion [19,20,21,22], underlining the strong glucose sensing capacity of HBP. These latter authors did not evaluate the HBP flux in function of the UDP-Glc*N*Ac amount generated upon glucose reduction, but measured intracellular protein *O*-Glc*N*Acylation, therefore it is not possible to exclude different mechanisms, such as OGT protein activity increase or stability.

Changes in glutamine availability in cell culture and tissues, as above described for glucose, also lead to a modulation of the UDP-Glc*N*Ac amount and HBP flux. In particular, in human cervix cancer cells and murine embryonic fibroblasts, the progressive glutamine increase from 0 mM to 8 mM causes a parallel increase in UDP-Glc*N*Ac (2.5-fold increase) or protein *O*-Glc*N*Acylation (almost 2-fold), respectively [14,23]. This glutamine-dependent effect on intracellular UDP-Glc*N*Ac concentration has also been observed in immune activated T cells upon addition of 2 mM glutamine, as compared to a complete depletion of glutamine from the medium [24]. The enhancement of UDP-Glc*N*Ac has also been observed in a more complex model, such as rat heart. Indeed, isolated rat hearts pre-treated with 2.5 mM glutamine show an ischemic protection associated with a significant increase in the levels of UDP-Glc*N*Ac and protein *O*-Glc*N*Acylation [25]. As previously described for glucose, for glutamine availability the effect on protein *O*-Glc*N*Acylation has also been well described. In fact, either in several diffuse large B-cell lymphoma cell lines or murine embryonic fibroblasts, an increased protein *O*-Glc*N*Acylation following the glutamine addition to cell culture has been observed [23,26], supporting the ability of HBP to adapt its flux in function of the intracellular glutamine availability.

UDP-Glc*N*Ac can also be generated through the salvage pathway, which requires the phosphorylation of Glc*N*Ac by Glc*N*Ac kinase (NAGK). Glc*N*Ac, the major component of complex carbohydrates found in glycoproteins, glycolipids, and proteoglycans, is thus recycled from the lysosomal degradation of these oligosaccharides. Some evidence suggests that this pathway complements the de novo synthesis of UDP-Glc*N*Ac [27,28]. The positive effect of the Glc*N*Ac supplementation on the intracellular UDP-Glc*N*Ac level has been observed in different cell and organism models. In mouse hepatocyte cells (AML12 cells), the addition of increasing concentrations of Glc*N*Ac (between 0 and 40 mM) leads to a six-fold increase in UDP-Glc*N*Ac [10]; similarly, in Jurkat T cells the supplementation of 10 mM Glc*N*Ac for 72 h increases the UDP-Glc*N*Ac almost six-fold [29]. This effect has been observed also in young male C57BL/6 mice, since oral Glc*N*Ac-supplementation also leads to an increase of intracellular UDP-Glc*N*Ac [10].

The HBP sensing for fatty acids has been poorly described. However, the few reports showed that in rat models the enhancement of free fatty acids by direct infusion induces an increase in UDP-Glc*N*Ac in their skeletal muscle [30] and that palmitate-stimulated myotubes respond with a 1.3-fold increase of UDP-Glc*N*Ac. These latter authors show also that UDP-Glc*N*Ac is enhanced by saturated C16 and C18 fatty acids, but not by unsaturated C16 and C18 fatty acids [31]. Even though the molecular mechanism of such an effect has not been well explained, these findings suggest a sensing role of HBP also for the intracellular fatty acid availability.

In this contest, the enhanced uptake and utilization of glucose and glutamine, as well as the increased fatty acid uptake and biosynthesis, at least in the specific context and tumor stage [32], may provide an explanation for the enhanced HBP flux observed in tumors [33,34,35,36].

On the other hand, the increase of HBP flux, UDP-Glc*N*Ac levels and protein glycosylation observed in cancer is also due to changes in the expression of the HBP enzymes. An important role for the sensing mechanism of the HBP, especially regarding glucose availability, must be assigned to the enzyme catalyzing the first step of the HBP, GFAT. It has been shown that an increased intracellular amount of glucose or of UDP-Glc*N*Ac may cause an enhancement or a reduction of GFAT activity, respectively [13,37,38,39]. Its important role in HBP flux has also been shown either by its chemical inhibition through the use of the glutamine analogues Azaserine and 6-diazo-5-oxo-l-norleucine (DON) [15] or by gene silencing [13]. Both approaches cause a significant depletion of the intracellular UDP-Glc*N*Ac pool, confirming its relevant role in HBP flux. GFAT is tightly controlled at the transcriptional level. In fact, diverse stimuli, among which some involved in cancer onset and growth, have been associated with GFAT gene expression regulation. For instance, *GFAT* mRNA increased expression has been observed upon EGF stimulation in breast cancer cells [40], upon androgen treatment in prostate cancer cells [41] and upon hypoxia in pancreatic cancer cells [42]. Conversely, oncogenic K-Ras inactivation in pancreatic cancer cells causes a significant down-regulation of *GFAT* mRNA and protein levels [43]. GFAT activity is controlled also by means of phosphorylation. Indeed, protein kinase A (PKA) and AMP-activated protein kinase (AMPK), as described below, are able to modulate its activity positively and negatively, respectively.

Altogether these findings indicate that HBP is able to adjust the flux in function of the glucose, glutamine and fatty acid availability and that this adjustment leads to a change of the level of the “sensor” molecule UDP-Glc*N*Ac. They also indicate that these nutrients might be substituted by Glc*N*Ac, through the action of NAGK, involved in the salvage pathway. In addition, they indicate an important role of the GFAT enzyme in HBP flux regulation. Finally, they indicate an association between HBP flux enhancement and cancer-specific metabolic changes.

## 3. Nutrient Transporters Function, Localization and Stability Is Regulated by HBP through the Control of Their Glycosylation Levels

As previously described, the main function of the HBP pathway is to generate the building blocks for glycoconjugate biosynthesis. Glycosylation is an important protein PTM occurring in the cytoplasm, the endoplasmic reticulum and the Golgi apparatus. *N*- and *O*-linked glycosylation, the major forms of eukaryotic glycosylation, have been identified in almost 50% of the cellular proteins. Abnormalities in protein glycosylation are common in several diseases, including cancer [44], and they can be a hallmark of carcinogenesis and cancer metastasis, since glycoconjugates play different roles in several steps of tumor progression, regulating tumor proliferation, invasion, metastasis and angiogenesis [4,33,44].

Among the different proteins involved in carcinogenesis and, in particular, in the metabolic rewiring observed in cancer, nutrient transporters carry out an important role. Accordingly, several oncogenic signaling pathways are strictly linked to the increased expression of the nutrient transporters [45,46]. Among the different mechanisms associated with nutrient transporter activity, more recently, their glycosylation status has been more investigated.

**Figure 2:**
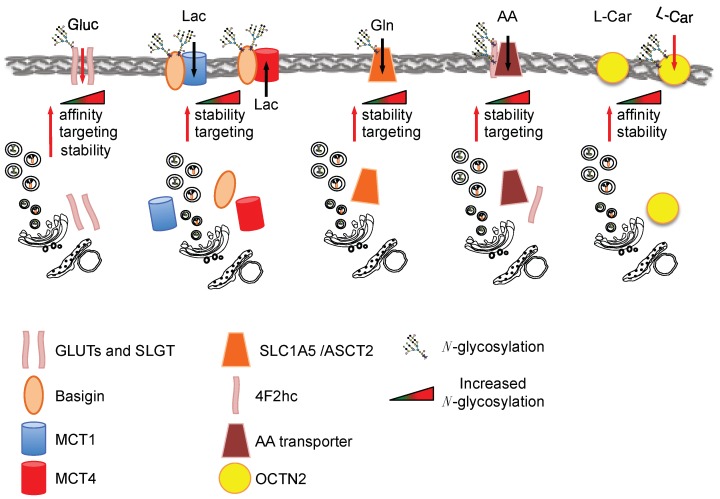
Schematic representation of the nutrient transporters discussed in the text. The arrows in the transporters indicate the direction of the flux. The red arrows indicate the increased nutrient transport and/or transporter affinity/targeting/stability. The single or double symbol of *N*-glycosylation indicates low or high glycosylated protein, respectively. The cartoon representing endoplasmic reticulum and Golgi indicates stability and targeting. Gluc: glucose; Lac: lactate; Gln: glutamine; AA: amino acid; l-Car: l-carnitine.

Glucose is an important nutrient for the proliferating cells and several glucose transporters are associated with cancerogenesis, such as the glucose transporter 1, 3 and 4 (GLUT1, GLUT3, GLUT4) and the sodium-dependent glucose transporter 1 (SGLT1) [47]. Previous studies showed that GLUT1 *N*-glycosylation is important for its transport activity [48], its membrane targeting and its stability [49]. K-Ras-transformed fibroblasts show an increased GLUT1 *N*-glycosylation associated with an enhancement of glucose uptake [50]. The importance of such *N*-glycosylation in different transformed cells has been shown using specific mutants of the glycosylation sites [49] and by using the *N*-glycosylation inhibitors tunycamicin and swainsonine [50,51,52]: both approaches supported the role of GLUT1 glycosylation for its targeting, stability as well as affinity for glucose. Further support for an association between GLUT1 glycosylation and its function has been gained in respiration-deficient cells, in which the enhanced glucose transport by GLUT1 is dependent on its glycosylation status [53]. Other glucose transporters involved in cancerogenesis, i.e., SLGT1, GLUT4 and GLUT3, have also been found to be glycosylated, but only a few reports have specifically addressed the role of glycosylation in their function [54]. Nevertheless, reduction of SLGT1 *N*-glycosylation lowers its affinity for the substrate [55] and the reduction of GLUT4 *N*-glycosylation reduces transporter stability [56]. Altogether these findings support a role of glycosylation in the function of this class of nutrient transporters (Figure 2). However additional work is needed to address the direct relation between the enhanced flux in HBP, glucose transporter activity, stability and localization specifically in cancer.

Another important class of nutrient transporters often overexpressed in cancer is monocarboxylate transporters (MCTs), which catalyze the proton-linked transport of monocarboxylates such as l-lactate, pyruvate and the ketone bodies [57]. Importantly, the MCT family does not appear to be glycosylated and its translocation seems to be dependent on the glycosylation of Basigin (CD147), a tumor-associated antigen highly expressed on the cell surface of various tumors [58] that is involved in the correct translocation of the transporters to the plasma membrane (Figure 2). Recently it has been shown that the mammalian target of rapamycin complex 2 (mTORC2), a protein kinase complex that plays a key role in nutrient sensing and cellular metabolism, is required to modulate the HBP via the regulation of GFAT1 expression. In fact, the inactivation of mTORC2 leads to profound defects in the metabolic pathways in association with the appearance of some under-glycosylated proteins, including CD147, suggesting a direct correlation between HBP, CD147 glycosylation and the metabolic changes observed in these mTORC2-inactiveted cells [59].

Another important metabolic change observed in tumors is the increased uptake and utilization of glutamine [60,61]. Accordingly, oncogenic transformation frequently up-regulates the expression of transporters such as the solute carrier family 1 member 5 (SLC1A5/ASCT2), a Na^+^-coupled amino acid transporter whose main function in cancer cells is glutamine uptake (Figure 2). Different structural studies have shown the presence of *N*-glycosylation sites in this transporter. In this regard, by using a mutagenic approach in the putative glycosylation sites, it has been recently suggested that the *N*-glycosylation of ASCT2 favors its delivery to the membrane and slows down its degradation. No effect on its activity has been observed [62]. The relation between HBP pathway and ASCT2 *N*-glycosylation may be argued from the results obtained by Polet et al. [63] in leukemia cells upon glucose depletion or tunicamycin treatment. Both treatments caused a significant de-glycosylation of ASCT2 in association with a significant mRNA up-regulation of other glutamine transporters (namely *ASCT2, SLC7A8, SLC38A1, SLC38A5, SLC7A5*), including *SLC7A5/LAT1*, which does not need glycosylation for its membrane localization, and thus appeared the most significant. Altogether these findings suggest that HBP flux may control glutamine and amino acid transporter membrane localization and stability.

Several other amino acid transporters have been found to be overexpressed in tumors, among which a relevant role in tumorigenesis has been indicated for SLC7A11 (xCT), LAT1, and the amino acid transporter dimerization partner 4F2 heavy chain antigen (SLC3A2/4F2hc). The 4F2hc is highly expressed in tumor cells and its expression correlates with tumor development, progression and metastatic potential [64]. In particular, in gastric cancer, in which it is overexpressed, it is only present in a highly glycosylated form as compared to normal tissue, in fact tunicamycin treatment of several gastric cancer cells leads to a reduction of its molecular weight from around 150 kDa to 60 kDa, the latter observed only in normal cells [65]. Only one study has addressed the role of the 4F2hc protein *N*-glycosylation. In fact, site-specific mutations of the three putative *N*-glycosylation sites causes protein half-life reduction [66]. A multiproteic CD147-4F2hc complex has been specifically identified on the proliferating cancer cell surface. This complex seems to play a critical role in energy metabolism, probably due to its capacity to coordinate either the transport of lactate (via MCT1 and MCT4) or amino acids (via LAT1 and xCT) [67,68].

Another transporter involved in the proliferation associated with specific metabolic changes of ER-positive breast cancer cells is the solute carrier family 22 member 5 (*SLC22A5*), which encodes an organic cation/carnitine transporter (also called OCTN2) [69,70]. This transporter has a high affinity for the carnitine directly involved in lipid metabolism and cellular bioenergetics [71]. OCTN2 *N*-glycosylation has an important role in its turnover and affinity for the substrate [72] (Figure 2).

Worthy of mention is also the effect of glycosylation on the different proteins involved either in lysosome function and/or autophagy. In fact, as reviewed elsewhere [73,74], the glycosylation of specific proteins, such as autophagy-related protein 9 (ATG9) (*N*-glycosylated) [75] and lysosome-associated membrane protein 1 and 2 (LAMP-1 and 2) (*O*- and *N*-glycosylated) [76,77], is directly involved either in the positive or negative regulation of autophagic flux, a process involved in both cancer suppression and promotion, depending on the cell context.

## 4. *O*-GlcNAcylation and Cancer

*O*-GlcNAcylation corresponds to the attachment of UDP-GlcNAc to specific Ser/Thr residues of target proteins. The *O*-GlcNAcylation is catalyzed by *O*-GlcNAc transferase (OGT). Conversely, the de-*O*-GlcNAcylation is catalyzed by β-*N*-acetylglucosaminidase (OGA) [7]. Protein *O*-GlcNAcylation influences either the activity or the stability of the target proteins and is involved in a complex interplay with phosphorylation, since these two PTMs can occur at the same or adjacent amino acidic residues, as well as at distant sites in the same protein [78]. As reviewed by Fardini et al., *O*-glycosylated proteins can be divided according to their function. In particular they can be grouped in proteins involved in the transcription and translation regulation (≈26%), in the cellular structure formation (≈14%), in the control of the cellular metabolism (≈13%), in the response to cellular stress conditions (≈11%), in the processes of protein folding and processing (≈7%), in cellular signaling (≈8%) and in cell cycle progression (≈2%) [79]. Considering the high number of cellular processes that are regulated by protein *O*-Glc*N*Acylation, an alteration of its level leads to profound changes in the cells.

The enhancement of protein *O*-Glc*N*Acylation has been observed in many tumors as a consequence of both an increase of the HBP flux, as described previously, and of the changes in the expression of the *O*-Glc*N*Acylation cycling enzymes, OGT and OGA (for review see [79,80,81]). Specifically, the increase of the OGT has been observed in different types of tumor, such as breast [82,83,84,85,86], colon [87,88], liver [89], endometrial [90], cervical [91], lung [87], prostate [41,92] and pancreatic [93] cancer, and it has often been associated with a parallel decrease of either the OGA levels or the OGA/OGT ratio [86,89,92]. Interestingly, such a change in the *O*-Glc*N*Acylation enzymes has been also correlated to the aggressiveness and the tumor grade [86,90]. Further, the increase in protein *O*-Glc*N*Acylation has been associated with the expression of an oncogenic K-ras protein [43], the activation of the MAPK/ERK signaling [94], the *c-Myc* amplification [95] and the tumor hypoxia [42]. Because these events, which influence either the HBP flux or OGT expression, are present in several types of tumors, protein hyper-*O*-Glc*N*Acylation, as described elsewhere [79,81], is more and more considered to be an important driver for tumor onset, progression, malignancy and appearance of cancer hallmarks.

Therefore, we will next describe its role in controlling cell metabolism and in particular the link with the metabolic rewiring observed in cancer cells.

## 5. *O*-GlcNAcylation of Metabolic Enzymes and Mitochondrial Proteins

**Figure 3:**
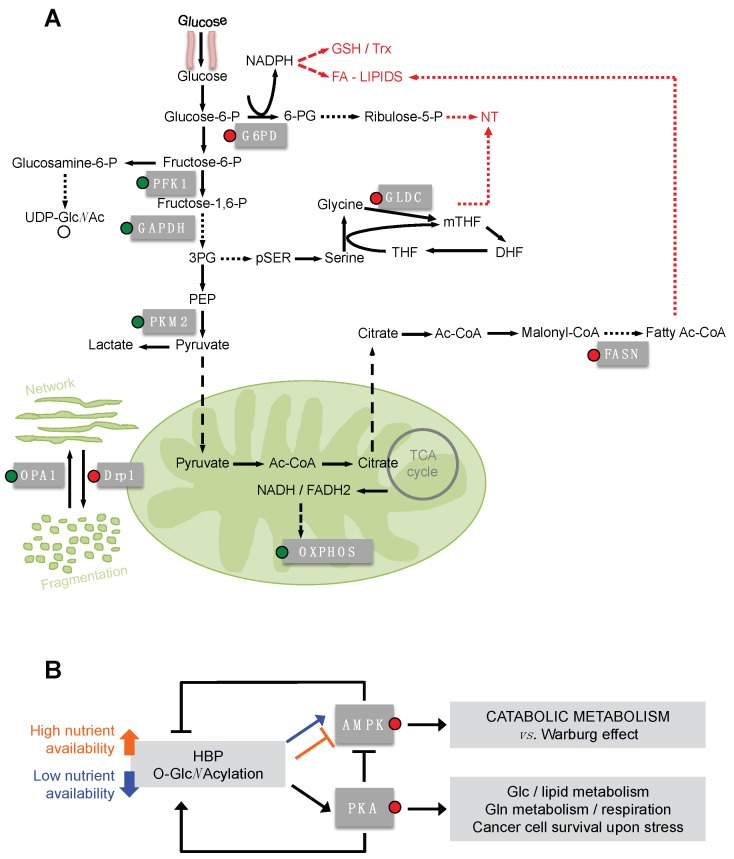
HBP modulates cancer metabolism through the *O*-Glc*N*Acylation of metabolic enzymes, signaling proteins and mitochondrial proteins. (**A**) In the image, the main metabolic pathways exploited by cancer cells for anabolism and bioenergetics are represented. The *O*-Glc*N*Acylated proteins (mainly the metabolic enzymes) are indicated in the grey boxes if the effect of the glycosylation has been demonstrated. The circle on the box, representing the *O*-Glc*N*Acylation, is red when the protein function is activated by glycosylation and green when it is inhibited. (**B**) The reciprocal regulation of AMPK and PKA and the HBP is schematically represented as discussed in the main text. 6-PG, 6-phosphogluconate; 3-PG, 3-phosphoglycerate; PEP, phosphoenolpyruvate; THF, tetrahydrofolate; DHF, dihydrofolate; FA, fatty acids; Fatty Ac-CoA, Fatty Acyl-Coenzyme A; GSH, glutathione; Trx, thioredoxin; NT, nucleotides.

Several authors have described the *O*-Glc*N*Acylation of different metabolic enzymes. In some cases the regulatory function of such a PTM in terms of protein localization, activity or function has also been clarified. Almost all glycolytic enzymes have been found to be *O*-Glc*N*Acylated [83,96,97,98] (Figure 3A) and for phosphofructokinase1 (PFK1), glyceraldheyde-3-phosphate (GAPDH) and pyruvate kinase M2 (PKM2) the effect of this PTM has also been investigated.

GAPDH is modified by *O-*Glc*N*Acylation mainly at Thr172. This modification favors the oligomeric instead of the tetrameric form of the enzyme, leading to its translocation into the nucleus and to the biological activities mediated by the non-tetrameric GAPDH [99]. Indeed, while the glycolytic activity is exerted by the cytoplasmic tetrameric GAPDH, the oligomeric nuclear form of the enzyme has roles in the transcriptional control of histone gene expression, fusion of the nuclear membrane, DNA repair and maintenance of the telomere structure [100]. Although the impact of the GAPDH *O-*Glc*N*Acylation on cell metabolism has not been deeply investigated, the functional redirection of the enzyme could lead to a decrease in the aerobic glycolysis, enhancing the flux through the glycolytic branches. This might be in accordance with the effect observed for PFK1 and PKM2 *O-*Glc*N*Acylation, which directly controls their glycolytic enzymatic activity, leading to important effects for cell metabolism. The *O-*Glc*N*Acylation of PFK1 at Ser529 inhibits its activity, blocking the binding of the allosteric activator fructose-2,6-bisphosphate (F-2,6-BP) and thereby impeding the F-2,6-BP-mediated association in higher oligomers with enhanced catalytic activity [101]. Such an *O-*Glc*N*Acylation-mediated inhibition of PFK1 activity leads to an increase of the PPP flux, reducing the glycolysis and the lactate production [101]. Interestingly, glucose-6-phosphate dehydrogenase (G6PD), the rate-limiting enzyme of the PPP, and phosphogluconate dehydrogenase (PGD), the enzyme that converts 6-phosphogluconate to ribulose-5-phosphate in the PPP, are also regulated by *O-*Glc*N*Acylation [97,98,102]. G6PD *O-*Glc*N*Acylation causes an increased affinity for NADP^+^ and favors its catalytically-active dimeric and tetrameric forms, consequently promoting the PPP [102]. Therefore it is evident that *O-*Glc*N*Acylation has a key role in regulating the flux of the PPP. In cancer cells, the increased level of HBP and *O-*Glc*N*Acylation leads to the up-regulation of the PPP flux, providing the cells with pentose sugars and NADPH for nucleotides and lipid biosynthesis, respectively, as well as for antioxidant glutathione and thioredoxin systems. Accordingly, the *O-*Glc*N*Acylation of PFK1 and G6PD promotes cancer cell proliferation and survival and provides a growth advantage to tumors in vivo [101,102]. *O-*Glc*N*Acylation also regulates PKM2, the main pyruvate kinase isoform expressed in cancer cells [103], leading to a decrease of its activity [104,105]. In particular, PKM2 *O-*Glc*N*Acylation at Thr405 and Ser406 destabilizes the tetrameric form, favoring the less active dimeric form [105]. Dimeric PKM2, upon the reduction of its activity, causes the accumulation of the upstream glycolytic intermediates that will enter in the glycolytic branches dedicated to cell anabolism. In addition, translocating into the nucleus induces *c-Myc* expression and consequently up-regulation of *GLUT1* and lactate dehydrogenase A (*LDHA)* mRNA, increasing the Warburg effect [105,106,107]. Therefore, in cancer cells, the enhanced HBP flux and consequently the higher level of UDP-Glc*N*Ac, leading to an increase of the *O-*Glc*N*Acylation of the glycolytic enzyme, represents an important mechanism for the regulation of the glycolysis, of the anabolic branches originated from glycolysis and of the maintenance of the Warburg effect [83,101,102,105]. It should be very interesting to discover the effect of the *O-*Glc*N*Acylation on the other glycolytic enzymes found glycosylated, such as HK1.

Correlated to the evidence of an *O-*Glc*N*Acylation-mediated increase of the cancer cell anabolism, some enzymes of the serine biosynthesis pathway, d-3-phosphoglycerate dehydrogenase (PHGDH) and phosphoserine aminotransferase 1 (PSAT1), of the glycine cleavage system, glycine decarboxylase (GLDC), and of thymidine synthesis pathway, thymidine kinase (TK), have also been found modified by *O-*Glc*N*Acylation [96,98,108,109]. Unfortunately, only GLDC *O-*Glc*N*Acylation has been studied in more detail. In particular, it has been shown that the increased expression of OGT, leading to GLDC *O-*Glc*N*Acylation, promotes migration and invasion in cervical cancer cells [108]. Since the increased expression of GLDC has been identified in many tumors and its metabolic activity has been associated with tumorigenesis, metastasis and poor prognosis [110,111,112,113,114,115], it is possible to speculate that the *O-*Glc*N*Acylation might favor the GLDC function and the pyrimidine biosynthesis.

It is worthy of note that the *O-*Glc*N*Acylation-mediated redirection of the metabolism to the anabolic branches of the glycolysis can also favor the flux through the HBP itself, establishing a positive loop. The competition between GFPT1 and PFK1 and between the aerobic glycolysis and the branching pathways has been clarified in T cells, where the high glycolytic flux limits the UDP-Glc*N*Ac biosynthesis as a consequence of the decrease of glucose and fructose-6-P available for HBP [116].

In addition to the glycolytic and the glycolysis-correlated pathways, lipid biosynthesis is also strictly regulated by *O-*Glc*N*Acylation through the modification of transcriptional factors, which will be subsequently discussed, and of some key enzymes. Recently, in a model of obese mice, a direct correlation between the expression and the activity of the fatty acid synthase (FAS) and its *O-*Glc*N*Acylation status has been identified [117]. In particular, the authors observed in these mice livers that the direct modification of FAS by *O-*Glc*N*Acylation increases its interaction with the deubiquitinating enzyme ubiquitin-specific protease-2A (USP2A) and prevents its degradation [117]. The USP2A-mediated stabilization of FAS has been indicated as a critical event for prostate cancer cell survival [118]. In addition, by using U2OS cells, Groves et al. have observed, in response to oxidative stress, a FAS-induced inhibition of OGA activity as consequence of an increased association between the two proteins [119]. All these elements suggest the existence of a regulatory loop that favors FAS activity in response to its *O-*Glc*N*Acylation.

*O-*Glc*N*Acylation has been identified in other metabolic enzymes such as pyruvate dehydrogenase (PDH) [96,120,121], isocytrate dehydrogenase (IDH), α-ketoglutarate dehydrogenase (OGDH), succinate dehydrogenase (SDH), malate dehydrogenase (MDH) [96,98,120,121], glutamate dehydrogenase (GLUD) and aspartate aminotransferase 1 and 2 (GOT1 and GOT2) [96,120,121]. Unfortunately, for these enzymes the effect of their *O-*Glc*N*Acylation has also not yet been investigated. However, considering that enhanced glutaminolysis may also lead to a reduced availability of glutamine entering in the HBP, it should be interesting to evaluate whether the *O-*Glc*N*Acylation has an effect on the enzymes involved in the glutamine utilization. In this regard, Tan et al. observed that the disruption of the *O-*Glc*N*Acylation cycling by overexpressing both OGA and OGT enzymes, reduced the levels of various TCA cycle proteins localized in the mitochondria [122]. In addition, *OGT* knock-out mice as compared to wild type showed enhanced muscle PDH and MDH basal activity, suggesting that a reduction of the intracellular level of *O-*Glc*N*Acylation leads to an activation of specific TCA cycle enzymes [123].

It is worthy of note that also many subunits of the electron transport chain (ETC) are *O-*Glc*N*Acylated [96,97,120,121,124,125,126]. While the role of such an *O-*Glc*N*Acylation in cancer is still under investigation, the aberrant *O-*Glc*N*Acylation cycling, as well as the silencing of OGT, have been analyzed either in normal cell physiology or in other human diseases. In particular, Dillmann’s group investigated the role of high-glucose-induced *O-*Glc*N*Acylation in cardiomyocites. They observed that the increased *O-*Glc*N*Acylation of the ETC proteins, including NDUFA9, UQCRC1, UQCRC2 and COXI, was associated with a lower activity of complex I, III and V and lower intracellular ATP levels and mitochondrial calcium [126]. In addition, they also observed that the *O-*Glc*N*Acylation also regulated the mitochondrial dynamics, since the increased *O-*Glc*N*Acylation of the fission protein dynamin-related protein 1 (DRP1) and of the fusion-related protein OPA1 promoted mitochondria fragmentation, associated with a decrease of the mitochondrial membrane’s potential, and more generally of the ETC activity [125,127]. Altogether these findings suggest that high levels of the mitochondrial proteins *O-*Glc*N*Acylation reduce mitochondrial function. On the other hand, in cardiomyocites, it has also been shown that an acute and brief overall increase of mitochondrial *O-*Glc*N*Acylation, induced by 12 h of treatment with the OGA inhibitor thiamet-G, has a positive effect on mitochondrial function, since an increased oxygen consumption and ATP production was observed [120]. Interestingly, in Alzheimer’s disease (AD), an association between mitochondrial protein *O-*Glc*N*Acylation and mitochondrial activity has also been observed. In fact the impairment of ATP synthase activity observed in AD appears associated with the loss of the interaction between the ATP synthase subunit a (ATP5A) and OGT, and consequently with a reduction of ATP5A *O-*Glc*N*Acylation at Thr 432, indicating that also in this model, the *O-*Glc*N*Acylation can sustain the OXPHOS activity through the regulation of Complex V [124]. Altogether these findings may suggest that the detrimental effect of *O-*Glc*N*Acylation on respiration might a consequence of the chronic alteration of HBP or OGT/OGA cycling, leading to constitutive mitochondrial protein *O-*Glc*N*Acylation. Conversely, the regulated process of mitochondrial protein *O-*Glc*N*Acylation may represent an important mechanism of mitochondrial function control, especially in physiological conditions. We would underline that although recent evidence, in contrast to the past assumptions born with Otto Warburg’s work, indicates that cancer cells need functional mitochondria for their growth and survival [128], it has been demonstrated that tumors present at least an attenuated respiration driven by oncogenes, like Ras, or environmental conditions, like hypoxia or hypoxia-like hypoxia-inducible factor 1-alpha (HIF1α) overexpression [129,130,131,132,133]. Interestingly, these are the same conditions that are responsible for the increased HBP flux and *O-*Glc*N*Acylation levels in cancer cells [42,43]. Thus, the aberrant *O-*Glc*N*Acylation might sustain the bioenergetics modification of tumors.

Taken together, all these observations indicate the protein *O-*Glc*N*Acylation as a shaper of cellular metabolism, pointing out the essential role that the HBP and *O-*Glc*N*Acylation cycling alteration could have in driving the remodeling of cancer metabolism and bioenergetics. Considering all the metabolic proteins found as *O-*Glc*N*Acylated, whose modification has not been yet investigated, this could be considered an important open research field.

## 6. *O*-GlcNAcylation of PKA and AMPK as Master Regulators of Cancer Cell Metabolism Downstream HBP Flux

Cell metabolism is controlled by master regulators, typically transcriptional factors or protein kinases, which control the function of numerous metabolic targets. Among these, AMPK and PKA seem to be part of a balanced regulatory loop involving the HBP (Figure 3B). AMPK is a sensor of the cellular AMP/ATP ratio, which in response to an intracellular increase of AMP or ADP levels switches the cellular metabolism from anabolism to catabolism in order to generate especially ATP [134]. In particular, in tumors AMPK negatively regulates the Warburg effect, inhibiting the lipid biosynthesis and mTOR-mediated HIF1α translation and stimulating the OXPHOS and the fatty acid oxidation [134,135,136,137]. PKA has a well-known role in regulating gluconeogenesis and lipid metabolism, mainly through the downstream transcriptional factor cAMP response element binding protein (CREB) [138,139,140] and has been identified as pivotal in controlling cancer cell metabolism in stress conditions, through the modulation of the glutamine metabolism, respiration and autophagy in glucose shortage and upon matrix-detachment [19,141]. A reciprocal regulation between the two kinases, AMPK and PKA, and the HBP has been shown. Indeed AMPK is able to inhibit GFAT activity upon phosphorylation. Such an inhibition reduces the detrimental cellular effects associated with hyperglycemia or the enhancement of intracellular protein *O-*Glc*N*Acylation, both correlated with specific human disorders [142,143]. Furthermore, AMPK phosphorylates OGT, influencing its localization and target selectivity [144]. In turn, the AMPK activity is controlled by the HBP flux through its *O-*Glc*N*Acylation. In this regard, Bullen et al. [144] showed that α-and γ-subunits of AMPK were *O-*Glc*N*Acylated by OGT in vitro and that this modification correlated with higher AMPK activity. Indeed, only Thr172 phosphorylated AMPK, the active form, was modified by *O-*Glc*N*Acylation, thereby demonstrating a selective and additive function of the two post-translational modifications. In addition, using skeletal muscle cells, they also observed that the activity of AMPK, induced by the nutritional and energetic stress upon glucose deprivation, was reduced in response to the treatment with the OGA inhibitor Thiamet G, which caused a strong increase in the cellular protein *O-*Glc*N*Acylation [144]. Note that the latter treatment mimics either a high HBP flux or the block of the *O-*Glc*N*Acylation cycling. Therefore, this and the other published data [144,145,146] suggest that when there is a high availability of nutrients and the flux through the HBP is elevated, the AMPK is kept off by the active metabolism and by the high level of ATP and the low AMP/ATP ratio. Conversely, when there is a nutrient shortage, AMPK is activated and the *O-*Glc*N*Acylation promotes its function. Thus, the HBP appears to be a sensor of the nutrient availability and one of the drivers of AMPK activation under nutrient shortage.

Conversely, PKA propels the HBP flux through the direct activation of the phosphorylation of GFAT [147,148] and, in addition, may favor autophagy and Glc*N*Ac recycling, especially under stress conditions [19]. The HBP activates PKA through the *O-*Glc*N*Acylation of its catalytic subunits, up-regulating the PKA-CREB signaling [149,150]. It is worthy of note that PKA is able to negatively regulate AMPK by phosphorylating the Ser173 residue and impeding the normal activating phosphorylation at Thr172 [151,152]. Thus, as shown in Figure 3B, PKA and AMPK might constitute a cycle favoring the maintenance of the HBP flux and the anabolic metabolism in rich nutrient conditions, through the AMPK inhibition that favors the activity of PKA over AMPK. In low nutrient availability (mainly glucose shortage) the balance between the two kinases, depending on the cell context, could represent a pivotal factor driving cell glycosylation and metabolism, growth and survival. Although these findings in great part have been obtained in other cell and disease models, if translated to cancer models they could open an interesting scenario of investigation.

## 7. Regulation of Cell Metabolism by *O*-GlcNAc Modified Transcriptional Factors

**Figure 4:**
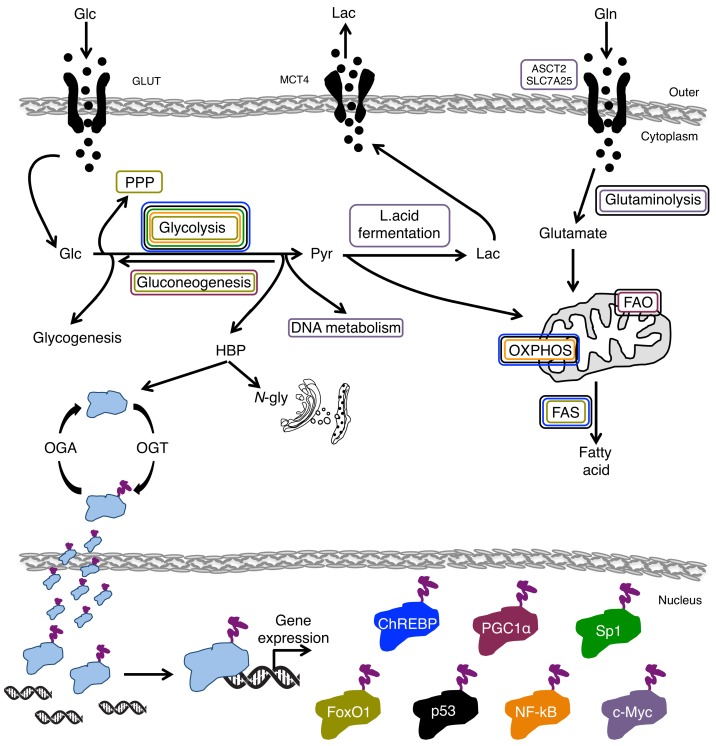
Schematic representation of the main metabolic pathways influenced (positively and/or negatively) by transcriptional factor *O-*Glc*N*Acylation. The figure depicts, from a schematic point of view, the transcriptional factors whose stability, transcriptional activation, DNA binding ability and protein–protein interaction is regulated by direct *O-*Glc*N*Acylation and the metabolic pathways influenced by their *O-*Glc*N*Acylation. The colored square around the indicated metabolic pathways is related to the specific transcriptional factor that upon *O-*Glc*N*Acylation is able to control the pathway.

As previously described, protein glycosylation may control several cellular functions. This section will focus attention on the *O-*Glc*N*Acylation of the transcriptional factor scenario and on its impact on the regulation of cellular metabolism (Figure 4). Here we subdivide transcriptional factors according to the effect of the *O-*Glc*N*Acylation on their stability, transcriptional activation, DNA-binding ability and protein–protein interaction.

### 7.1. Protein Stability

#### 7.1.1. c-Myc

The stability of the oncogenic transcription factor c-Myc is regulated by phosphorylation and *O-*Glc*N*Acylation at Thr58, present in its transactivation domain (TAD); the aforementioned PTMs are mutually exclusive [153]. Specifically, the *O-*Glc*N*Acylation at Thr58, preventing phosphorylation by GSK3β, blocks c-Myc ubiquitination and proteosomal degradation [154], enhancing its activation.

c-Myc is one of the master regulators of cell metabolism due to its ability to activate the expression of several genes involved in nucleotide, glucose and glutamine metabolism and in the control of mitochondrial and ribosome biogenesis. Indeed, c-Myc promotes the gene expression of the ornitine decarboxylase (*ODC*) [155], carboamyl-phosphate synthase (*CAD*) [156], dihydrofolate reductase (*DHFR*) [157] and thymidine kinase (*TK*) [158], which are all involved in DNA metabolism. In normoxia, c-Myc activation enhances the expression of some glycolytic genes such as *HK2*, *PFK-M*, *ENO1* [159] and *LDH-A* [160], and is able to confer glutamine addiction by directly up-regulating the glutamine transporters *ASCT2* and *SLC7A25* and indirectly up-regulating enzyme glutaminase (*GLS*) through the inhibition of miR-23 [161]. Glutamine can be then oxidized in the TCA cycle to generate high-energy electrons and the production of ATP, it can undergo glutaminolysis, also constituting the substrate for amino acids and GSH synthesis, and it can fuel the HBP [162].

Since its involvement in different metabolic pathways, the association between de-regulated c-Myc in tumors, the stability of which is increased upon *O-*Glc*N*Acylation, and the Warburg effect, is arguable. Accordingly, it has been reported that in human prostate cancer, a synergy between elevated *O-*Glc*N*Acylation levels and *c-Myc* locus amplification occurs, leading to increased glycolysis [41]. In addition, hyper-*O-*Glc*N*Acylation and c-Myc overexpression have been associated with cisplatin resistance in human lung carcinoma [163].

#### 7.1.2. Peroxisome Proliferator-Activated Receptor-Gamma Coactivator-1α

Peroxisome proliferator-activated receptor-gamma coactivator-1α (PGC-1α) belongs to a family of transcriptional co-activators involved in cellular energy metabolism, considered the master regulator of gluconeogenesis. In addition, for PGC-1α a key role in mitochondrial respiration, detoxification of reactive oxygen species and fatty acid oxidation has also been reported [164].

According to the role of the altered glucose homeostasis in determining the onset of diabetes, a mechanism has been described by which the gluconeogenic genes are highly transcribed upon the direct interaction of PGC-1α with a complex formed by OGT and the co-activator human factor C-1 (HCF-1) [165]. This interaction causes PGC-1α *O-*Glc*N*Acylation on Ser333, preventing its degradation [166]. This stabilization leads to an enhancement of the PGC-1α-dependent transcription of the gluconeogenic genes, glucose 6-phosphatase (*G6Pase*) and phosphoenolpyruvate-carboxykinase (*PEPCK*). Similar results have also been obtained in a mouse model of diabetes, the *db/db* mice [165]. Indeed, in these mice the knocking-down *OGT* and/or *HCF-1* genes, leading to the disruption of the OGT/HCF-1 complex, caused an improvement of the disease [165]. The *O-*Glc*N*Acylation of PGC-1α has been also involved in the regulation of thermogenesis in brown adipose tissue [167]. In fact, PGC-1α *O-*Glc*N*Acylation is also associated with an increase in fatty acid oxidation [168] by the direct modulation of the expression of ETC genes [167] in association with some other transcriptional factors or co-activators (NRF1, ERR, YY1, MEF2C, PPAR) [168]. The association between PGC-1α *O-*Glc*N*Acylation and its stability has been indirectly demonstrated by using an activating point mutation (C430S) in the porcine PGC-1α [169]. This mutant showed an increased level of *O-*Glc*N*Acylation associated with higher protein stability and activity as compared to wild type protein [169]. It is important to note that PGC-1α activity has been linked to different metabolic alterations of cancer cells [164]. For instance, the formation of a complex formed by PGC-1α, MITF and ERRα, observed in some melanoma and breast tumors, induces an enhancement of the OXPHOS associated with major therapeutic resistance and to anchorage-independent cell growth [164]. A role of PGC-1α in the activation of mitochondrial biogenesis has been also demonstrated in circulating breast tumor cells promoting metastasis [170]. In addition, some studies have also highlighted a role of PGC-1α either in the enhancement of fatty acid oxidation observed in breast [171] and prostate tumors [172], or in the activation of lipogenesis in colon cancer and in ERBB2/Neu-induced breast cancer [167,173,174].

In conclusion, the *O-*Glc*N*Acylation of PGC-1α increases its stability and enhances its activity, leading to the activation of different cancer-specific metabolic pathways.

#### 7.1.3. Tumor Suppressor p53

The tumor suppressor p53 regulates cell growth and apoptosis under several cellular stress conditions through its transcriptional factor activity [175]. p53 protein stabilization and activity depend on several PTMs that regulate its ubiquitination and proteosomal degradation [176]. Recently, p53 has been defined as a sensor of the intracellular *O-*Glc*N*Acylation level, since it is regulated according to the intracellular protein *O-*Glc*N*Acylation level [177]. Mass spectrometry analysis revealed that the *O-*Glc*N*Acylation of the Ser149, localized near Thr155, is able to prevent the phosphorylation of Thr155, increasing the p53 stabilization and nuclear translocation [178]. de Queiroz et al. have demonstrated that an increase of cellular protein *O-*Glc*N*Acylation promotes p53 acetylation at Lys382, increasing p53 DNA binding and the activation of its target gene [177]. Altogether, these findings suggest that p53 *O-*Glc*N*Acylation regulates p53 stability and activity. In addition, by using a site-directed mutagenesis approach, it has been suggested that p53 *O-*Glc*N*Acylation may occur in other undefined residues, since the mutation of Ser149 does not completely abolish p53 *O-*Glc*N*Acylation [178]. Nevertheless, the role of these other residues in p53 function has not yet been defined.

It is worthy of note that the role of p53 in regulating cellular metabolism is well described. Indeed, through its transcriptional activity, it negatively regulates glycolysis and lipid biogenesis and positively regulates oxidative phosphorylation, glutaminolysis, and fatty acid oxidation (FAO) [179]. This metabolic regulation correlates with its oncosuppressive function, since it counteracts the metabolic rewiring observed in cancer cells, such as the Warburg effect. Most likely, p53 *O-*Glc*N*Acylation, by controlling its activity, is involved in the transcriptional activation of other metabolic targets associated with its oncosuppressive function, however no investigation has been conducted on this particular aspect of p53 regulation in cancer.

### 7.2. Transcriptional Activity

#### Forkhead Box Other-1

Forkhead box other-1 (FoxO1) is a forkhead box family member, able to control the expression of genes involved in the regulation of the cell cycle, in response to oxidative stress and apoptosis. FoxO1 appears to be a highly *O*-Glc*N*Acylated, since at least five different residues have been found to be modified by this PTM (Thr317, Ser550, Thr648, Ser654 [166] and Thr646 [180]). In addition, upon abnormal *O-*Glc*N*Acylation, often observed in chronic hyperglycaemia patients, FoxO1 promotes the transcription of *PGC-1α*, which in turns interacts with FoxO1, in order to activate the genes involved in hepatic gluconeogenesis, *G6Pase* and *PEPCK*, leading to further glucose production and therefore to the enhancement of FoxO1 *O-*Glc*N*Acylation [181]. Although increased FoxO1 *O-*Glc*N*Acylation correlates with its increased transcriptional activity, functional studies have indicated that only the mutation of the Thr317 residue slightly reduces FoxO1 transcriptional activity [180].

FoxO1 generally functions as a tumor suppressor in cancer [182]. Emerging evidence, indeed, reveals that FoxO1 represses glycolysis and mitochondrial respiration by inhibiting c-Myc and thus counteracting the Warburg effect [183]. Moreover, FoxO1 may negatively regulate SREBP1c-mediated fatty acid and cholesterol biosynthesis, which are up-regulated in cancer [184], by occupying and disrupting the assembly of the transcriptional initiation complex on the SREBP1c promoter [185].

The findings indicate that the *O*-Glc*N*Acylation of the two tumor suppressors FoxO1 and p53 enhances their activity; this is rather counterintuitive to the fact that cancer increases the level of protein *O*-Glc*N*Acylation. However, it is important to underline that both p53 and FoxO1 are inactivated by mutation in almost all tumors or in several types of tumors, respectively [186,187,188]. Therefore, the *O*-Glc*N*Acylation of these two proteins may be involved in normal cell metabolism, as previously described, in other types of human diseases, as well as at an early phase of tumor development. In the near future it will be interesting to investigate the role of tumor suppressors *O*-Glc*N*Acylation in correlation with the metabolic changes observed in cancer, especially at the early onset.

### 7.3. Protein–Protein Interaction

#### Nuclear Factor Kappa-Light-Chain-Enhancer of Activated B Cells

Nuclear factor kappa-light-chain-enhancer of activated B cells (NF-κB) is another important transcription factor involved in cancerogenesis, the activity of which is regulated by *O-*Glc*N*Acylation. For instance, in pancreatic ductal adenocarcinoma (PDAC), the mutation of the two *O-*Glc*N*Acylated sites of the NF-κB-p65 subunit, Thr352 and Th322, causes a dramatic decrease of cancer cell growth [93]. Notably, it has been reported that the *O-*Glc*N*Acylation of these two residues in NF-κB-p65 causes a decreased binding to the IkBa protein (the inhibitor of NF-κB), enhancing the NF-κB stability, and causing increased transcriptional activity [189]. Furthermore, in PDAC, it has been also shown that the *O-*Glc*N*Acylation of IKKα, a subunit of the kinase (IKK) that positively regulates NF-κB activity, is associated with an enhancement of its kinase activity that leads to the increased stabilization and nuclear translocation of NF-κB [93]. NF-κB’s ability to regulate cell metabolism, acting both on glycolysis and OXPHOS, has also been reported [190]. In particular, NF-κB metabolic regulation depends on the p53 genetic status. Indeed, in p53-deficent cancer cells the active NF-κB is able to enter into mitochondria [191], where it suppresses mitochondrial gene expression and OXPHOS. Conversely, promoting nuclear *GLUT3* gene expression boosts glycolysis [192]. Notably, this increased GLUT3 expression leads to enhanced glucose uptake and HBP flux that further induces NF-κB activity, promoting IKK stabilization through Ser733 *O-*Glc*N*Acylation. Altogether, these observations suggest that the cancer metabolic rewiring is favored by the existence of a positive feedback loop between IKK-NF-κB *O-*Glc*N*Acylation and the enhancement of the glycolysis [192].

### 7.4. DNA Binding

#### Carbohydrate-Responsive Element Binding Protein

Carbohydrate-responsive element binding protein (ChREBP) is a key regulator of glycolysis and lipid metabolism through its capacity to act as a transcriptional activator of both glycolytic and lipogenic genes [193,194], as well as L-pyruvate kinase (*L-PK*) [195], Ac-CoA carboxylase (*ACC*) and *FAS* [196].

ChREBP interacts directly with OGT [197]. Although the precise protein location of the *O-*Glc*N*Acylated residues has not been yet identified, it has been clearly shown that they are in a different position and have an opposite function with respect to the phosphorylated sites [197]. For instance, the PKA-dependent phosphorylation of ChREBP, which promotes its interaction with the 14-3-3 protein, retains the ChREBP cytosols in an inactivated status [197]. Conversely, ChREBP *O-*Glc*N*Acylation enhances its activity and the binding to the carbohydrate response elements (ChoRE) in the promoter region of specific metabolic target genes [198]. Although a large amount of data highlights the key role of ChREBP in hepatocytes, a growing body of literature also reveals the involvement of ChREBP in cancer. Indeed, recent studies delineate a role of ChREBP, through its transcriptional activity, in tumor initiation and in the progression of different solid and non-solid tumors [199]. A connection between ChREBP function and tumor metabolic reprogramming has also been well-established [200]. Indeed, upon ChREBP suppression, HCT116 colorectal cancer cells and HepG2 hepatoblastoma cells show a reduction of aerobic glycolysis, of lipogenesis and of nucleotide biosynthesis; conversely a concomitant increase in oxygen consumption through the activation of mitochondrial respiration has been observed. All these metabolic features correlate with decreased tumor potential [200,201].

### 7.5. Subcellular Localization, Stability and Transcriptional Activity

#### Specificity Protein 1

The zinc finger transcription factor specificity protein 1 (Sp1) is the first transcription factor identified as *O*-Glc*N*Acylated [202]. Sp1 regulates cell survival, growth and angiogenesis by activating the transcription of specific target genes, and its abnormal expression is found to be associated with cancer development and major aggressiveness [203]. Sp1 subcellular localization, stability and transcriptional activity are regulated by PTMs, including *O-*Glc*N*Acylation [204]. The analysis of the Sp1 *O-*Glc*N*Acylation status has permitted the identification of at least eight *O*-Glc*N*Acylated residues [205]. These sites, identified in the zinc finger DNA-binding domain and in the domain necessary for the interaction with other transcription factors or co-activators, control either the Sp1 DNA binding capacity or its basal transcriptional activity [206,207]. Nevertheless, Sp1 *O-*Glc*N*Acylation, as indicated in different reports, has been associated either with negative or positive transcriptional activity with regard to cell metabolism [202,208,209]. For instance, it has been shown in HEK293 cells, that Sp1 *O-*Glc*N*Acylation leads to the negative transcriptional regulation of the glycolytic genes. Indeed, the expression of *O-*Glc*N*Acylation mutants of Sp1 causes the up-regulation of several mRNA encoding for glycolytic enzymes, such as *PFK-L* and *PFK-M*, triose phosphate isomerase (*TPI*), *GAPDH*, phosphoglycerate kinase1 (*PGK1*), phosphoglycerate mutase 1 (*PGAM1*), *ENO1* and *PK-M* [210]. Penque et al. have shown that in the hyperinsulinemic condition, the activation of Sp1 by *O-*Glc*N*Acylation is associated with a cholesterolgenic response in murine 3T3-L1 preadipocytes. Indeed, *O*-Glc*N*Acylated Sp1 induces the expression of the sterol response element-binding protein 1 (*SREBP1*) and of 3-hydroxy-3-methyl-glutaryl-coenzyme A reductase (*Hmgcr*), which increase the amount of plasma membrane cholesterol, and negatively affect the correct GLUT4 membrane localization [211,212,213]. Another example of the relation between Sp1 *O-*Glc*N*Acylation and metabolism has been observed in the Caco-2/TC7 cell line (intestinal enterocytes) upon cell treatment with glutamine or glucosamine. Both treatments, increasing Sp1 *O-*Glc*N*Acylation, permit the transcriptional activation of the argininosuccinate synthase (*ASS*) gene [214]. This observation underlines the important role of Sp1 in cancer metabolism. Indeed, it has been reported that arginine depletion, a consequence of the *ASS1* silencing in several cancer cell lines, causes a reduction in aerobic glycolysis and a parallel increase in serine biosynthesis, glutamine anaplerosis and oxidative phosphorylation [215].

The involvement of Sp1 in cancer metabolic rewiring has been further shown in different reports. Sp1 activation in colon and prostate cancers is associated with the activation of several genes involved in fatty acid synthesis, such as *FAS* [216], ATP citrate lyase (*ACLY*) and Acetyl-CoA carboxylase (*ACACA*) [217]. In addition, Sp1 expression and activity has also been associated with the overexpression of the human testis-specific lactate dehydrogenase c gene (*hLDHC*) in lung, melanoma and breast cancer [218,219]. Importantly, the abnormal activation of Sp1 has been identified in several types of tumors such as pancreas [220], gastric [221] and epithelial [222]. According to these data, Sp1 *O-*Glc*N*Acylation, increasing its activity and stability, may be directly involved in the cancer metabolic rewiring associated with cancer onset.

In conclusion, all aforementioned reports highlight the pivotal role of *O-*Glc*N*Acylation and hence of HBP flux in controlling cell metabolism via the regulation of the key transcription factors involved either in the regulation of physiological cell metabolism or, when deregulated, in human diseases such as cancer.

## 8. Concluding Remarks

Tumor metabolic reprogramming is considered one important cancer hallmark. For this reason, recently more and more researchers have addressed their studies to the identification of these cancer-specific metabolic features as well as to the definition of their role in tumor onset, maintenance and aggressiveness. The overwhelming number of findings, shedding new light on cancer metabolism, has also unlocked the possibility of targeting the metabolism for cancer therapy. Among the different metabolic pathways identified in cancer, an important role has been assigned to the HBP. In fact, the HBP, as described in this review, may be considered the only metabolic route able to continuously monitor the cellular nutrient status and to generate a “signaling” metabolite, the end product UDP-Glc*N*Ac, able to lead to a fine adjustment of the cellular metabolism in direct correlation with nutrient variations. Such a prominent role in controlling cell metabolism has linked its alterations to the etiology of different human diseases such metabolic syndromes, i.e., diabetes [223], neurodegenerative diseases [224], aging [225] and cancer. Regarding cancer, we reviewed how this metabolic pathway may be involved in the metabolic reprogramming of cancer cells. Indeed, first, we defined its role as a nutrient sensor, describing how the main intracellular metabolites, namely glucose, amino acids and fatty acids are able to influence the HBP flux. Second, we described how the change of the HBP flux influences nutrient transporter expression, stability, affinity for the substrate and membrane targeting, through the modulation of their *N*-glycosylation status. Third, we emphasized the positive and negative role of *O-*Glc*N*Acylation in the regulation of glycolysis, PPP, fatty acid metabolism and mitochondrial activity, either by the direct PTM of the different proteins involved in the above metabolic pathways, or indirectly, through the *O-*Glc*N*Acylation of transcriptional factors acting in cancer cell metabolism rewiring.

One interesting extension of these observations is that HBP may be considered the main cellular nutrient sensor pathway. In fact, compared with other sensor pathways, such as AMPK and mTOR, which act in response to a reduction of ATP or to an alteration of the amino acid levels, respectively, the HBP senses all the main types of intracellular nutrients leading to an orchestrated metabolic response. In addition, whilst our understanding of nutrient sensing mechanisms is far from complete, we can also argue that HBP is the most rapid and upstream metabolic pathway able to respond to nutrient variations. In fact, the HBP flux that is modulated by either a surplus or a shortfall of nutrients must be on the top of the sensing signaling cascade in such a way as to rapidly modulate cellular bioenergetics and metabolism.

Given this important role in metabolism, it does not appear surprising that in cancer cells this pathway is often up-regulated either in terms of gene and protein expression or in terms of flux. As we reviewed, its activation entirely resembles the most important metabolic alterations observed in tumors, such as increased glycolysis, increased glutamine utilization, increased fatty acid synthesis as well as great changes in mitochondrial bioenergetics. In our opinion, in the near future studies regarding the connection between HBP and cancer will increase, since several points still need to be addressed. For example, it will be useful to have tumor/cell profiles of protein glycosylation under normal growth conditions and nutrient perturbed conditions, which may help to define the glycosylation signatures associated with the cancer’s ability to adapt to nutrient variations. Furthermore, the identification of the other transcription factors will be useful, with particular attention dedicated to the oncogenic factors whose *O-*Glc*N*Acylation may alter cell metabolism. It will be useful as well to investigate the role of nutritional load or nutrient processing, for instance the role of bacterial flora, on the HBP activation and hence in the development of cancer, i.e., colon cancer. Finally, it will be useful to identify and validate novel putative HBP inhibitors either as tools for a deeper understanding of this pathway or as new drugs for cancer therapy. The latter is a tremendously interesting field, since HBP is involved in all the cancer hallmarks [226]. In fact, as some recent publications clearly highlighted [26,227,228], the inhibition of HBP or one of its two branches, controlling *N*-glycosylation or *O-*Glc*N*Acylation respectively, may help to develop new therapies for human cancer. Undoubtedly, these approaches will bring a substantial value to the field of cancer metabolic rewiring, but, in perspective, they could also be applied to other human diseases, such as neurodegeneration or aging.

We wish to apologize to all the authors who were not cited in this review. We tried to identify all the references linked to the subject of the review, but probably missed some authors. However we believe that the articles reviewed are good examples supporting a role of HBP in cancer metabolism rewiring.
